# Amelioration of Experimental Autoimmune Uveitis by Leflunomide in Lewis Rats

**DOI:** 10.1371/journal.pone.0062071

**Published:** 2013-04-23

**Authors:** Cheng-bo Fang, De-xi Zhou, Shu-xiang Zhan, Yong He, Zhen Lin, Cheng Huang, Jun Li

**Affiliations:** 1 School of Pharmacy, Anhui Key Laboratory of Bioactivity of Natural Products, Anhui Medical University, Hefei, China; 2 Department of Ophthalmology, The 1st Affiliated Hospital of Anhui Medical University, Hefei, China; Oregon Health & Science University, United States of America

## Abstract

**Purpose:**

To investigate the efficacy of leflunomide in experimental autoimmune uveitis (EAU) in rats.

**Methods:**

Lewis rats were immunized with interphotoreceptor retinoid-binding peptide (IRBP) in order to generate EAU. Rats received three dose of leflunomide through intragastric administration (prevention or treatment protocols) after immunization at three separate doses (3 mg/kg/d; 6 mg/kg/d; 12 mg/kg/d). Cyclosporin A was administered as a positive) control. Rats were euthanized during peak disease activity (day 14 or 15). Treatment effectiveness was evaluated *in vivo* using clinical EAU scoring (d14) and histopathological evaluation of enucleated eyes after experimental termination. The expression levels of inflammatory cytokines in the serum were quantified by ELISA. Eyeball of rats were harvested and mRNA expression of interleukin 17 (IL17) and IFN-γ were quantified through RT-PCR. Intracellular expression of interleukin (IL)-17 in the activated CD4(+) T cells was assessed by flow cytometry. The effects of leflunomide inhibition on immune responses in rats were investigated in isolated lymphocytes.

**Results:**

Histopathological and clinical data revealed severe intraocular inflammation in the immunized rat. Inflammation reached its peak on day 14 in this EAU model. Treatment with leflunomide significantly prevented and treated EAU-induced ocular inflammation and decreased clinical and pathological scores compared to vehicle-treated eyes. Gene expression of IL17 and IFN-γwas markedly reduced in leflunomide-treated eyes. Leflunomide significantly decreased the serum levels of IL17 and IFN-γ. The study of IL17+ T cells in peripheral blood and spleen by flow cytometry showed a decreased number of Th17 cell in rats of leflunomide prevented group. Lymphocytes from animals treated with leflunomide had decreased antigen-specific proliferation *in vitro* compared with lymphocytes from untreated animals.

**Conclusions:**

Oral administration of leflunomide effectively suppressed IRBP-induced uveitis in rats. These results suggest that leflunomide may be potentially clinical application in uveitis.

## Introduction

Uveitis, which is defined as inflammation of the middle vascular layer of the eye, is one of the most common causes of blindness and visual impairment worldwide [1]. Human autoimmune uveoretinitis is thought to be caused either by an autoimmune response or by an unknown etiology [2], [3].

Experimental autoimmune uveitis (EAU) is an animal model representing human autoimmune uveitis. This experimental model is useful for determining the cause of human posterior uveitis and for developing new therapeutic strategies [4], [5], [6]. EAU is predominantly a T-cell-mediated disease. A Th1 response is thought to be an essential factor in EAU pathogenesis. Recent evidence suggests that newly recognized interleukin (IL)-17, produced by T helper IL-17 cells, plays a crucial role in the disease progress of EAU, and that a Th1 and Th17 response are differentially required for EAU development [7], [8], [9], [10], [11].

Leflunomide (LEF), a new disease-modifying antirheumatic drug of the isoxazol family, is clinically used in the treatment of rheumatoid arthritis, sarcoidosis, solid organ transplantation, lupus nephritis and the course of several autoimmune diseases [12], [13], [14]. Korn et al. gave leflunomide orally at 20 mg/kg per day completely protected the rats from clinically apparent EAN [15]. The primary metabolite of leflunomide reversibly inhibits dihydroorotate dehydrogenase, which leads to diminished DNA synthesis and impaired proliferative capacity [16]. Unlike other cells, activated lymphocytes expand their pyrimidine pool by eight to sixteen-fold during proliferation and must use both salvage and *de novo* pathways of synthesis to meet this metabolic demand. In the presence of leflunomide, T cell proliferation is inhibited [17], [18]. By decreasing the supply of pyrimidines, leflunomide leads to the interruption of the cell cycle and decreased proliferation of activated lymphocytes. Therefore, leflunomide (LEF) is an interesting potential therapeutic agent in the treatment of EAU.

Despite its known immunomodulatory effects, there are very few studies regarding the role of LEF in uveitis [19], [20], [21]. However, in animal studies administration of leflunomide resulted in the inhibition of IFN- γ production [22], [23]. In this study, the efficacy of leflunomide was determined in the rodent EAU model. Leflunomide strikingly ameliorated both the clinical symptoms and the pathologic manifestations during peak phases of the disease. We report the successful use of leflunomide for the prevention and treatment of EAU and the effective inhibition of Th17 responses in Lewis rats.

## Methods

### Animals

Female Lewis rats, 6–8 weeks old, were purchased from Vital River (Beijing, China) and housed in SPF conditions for 1 week prior to experimentation. The animals had access to food and water *ad libitum*. All animal experiments were approved by the Laboratory Animal Care and Use Committee of the Anhui Medical University and adhere to the ARVO Statement for the Use of Animals in Ophthalmic and Vision Research.

### Drugs and Reagents

The interphotoreceptor retinoid binding protein peptides spanning amino acid residues 1169–1191 (IRBP_1169–1191,_ PTARSVGAADGSSWEGVGVVPDV) were synthesized and purified by Shanghai Sangon Biological Engineering Technology & Services Ltd. Co. Complete Freund’s Adjuvant, Pertussis toxin Ficoll-Hypaque gradient reagent, and 3-(4,5-dimethylthylthiazol-2-yl)-2,5-diphenyltetrazolium bromide (MTT) powder were purchased from Sigma-Aldrich Chemical (St. Louis, USA). Leflunomide was purchased from Cinkate Pharmaceutical (Shanghai, China). RPMI-1640 medium (pH adjusted to 7.2) was purchased from Gibco (USA) and was supplemented with 25 mmol/L Hepes, 2 mmol/L L-glutamine, 50 µmol/L 2-mercaptoethanol, 100 IU/L penicillin sodium, 100 IU/L streptomycin, and 1 mmol/L sodium pyruvate. All other chemicals and solvents were purchased from local firms (Hefei, China) and were at the highest purity and analytical grade.

### Induction of EAU and Treatment Protocols

On day 0, Lewis rats were immunized with 100 µg IRBP peptide emulsified in CFA. The emulsion was injected into one hind footpad in a total volume of 0.1 ml and Pertussis toxin (400 ng) was given intraperitoneally as an additional adjuvant.

Leflunomide, in powder form, was dissolved in 0.5% carboxymethyl cellulose. Cyclosporin A (CsA) was solubilized in olive oil at 25 mg/mL. Therapeutic agents were leflunomide (administered in low [3 mg/kg], medium [6 mg/kg], and high [12 mg/kg] doses), CsA (positive control, 10 mg/kg), and vehicle (negative control, physiological saline). Rats were divided into two main treatment groups, preventive and therapeutic. Preventively treated rats were divided into six treatment groups. Treatment with leflunomide in a volume of 1.0 ml carboxymethyl cellulose was given orally by gavage from day (d) 1 to d13 post-immunization. Cyclosporin A was gavaged at 10 mg/kg from d1 to d13 post-immunization. Negative controls were gavaged with physiological saline only. Treatments in the therapeutic rats were delivered daily in the same manner, but treatments began from d8 with respect to IRBP immunization. Each treatment group consisted of six to eight rats. Experiments were performed twice.

### Clinical Evaluation of EAU and Scoring

Clinical assessment by slit lamp microscopy examination of ocular inflammation was conducted every day from day 7 after immunization. Each eye was graded, without masking, according to a previously described scoring system: 0, normal; 0.5, dilated blood vessels in the iris; 1, abnormal pupil contraction; 2, hazy anterior chamber; 3, moderately opaque anterior chamber with dull red reflex; 4, opaque anterior chamber, absent red reflex, and proptosis [24].

### Histopathological Evaluation of EAU and Scoring

Animals were euthanized during peak disease activity (day 14 or 15), and the eyes were enucleated, fixed in 4% buffered paraformaldehyde, embedded in paraffin, sectioned (4–6 µm) through the papillary-optic nerve plane, and stained with hematoxylin and eosin. Presence or absence of disease was evaluated blindly by examining six sections sectioned at different levels for each eye. Severity of EAU was scored on a scale of 0 (no disease) to 4 (maximum disease) in half-point increments based on the presence of inflammatory cell infiltration of the iris, ciliary body, anterior chamber, and retina (0 = normal anterior segment and retinal architecture, with no inflammatory cells in these structures; 1 = mild inflammatory cell in filtration of the anterior segment and retina; 2 = moderate inflammatory cell infiltration of the anterior segment and retina; 3 = massive inflammatory cell in filtration of the anterior segment and retina, disorganized anterior segment and retina; and 4 = as in 3 but with photoreceptor cell damage) [25], [26]. No information about the experimental design or treatments was provided to the pathologist.

### Lymphocyte Proliferation Assay and In Vitro Suppression

At the termination of the experiment on d14 after immunization, spleens and draining lymph nodes were harvested. The lymphocytes were prepared by pushing spleens through a 100 µm nylon mesh. Lymphocytes from spleen and draining lymph node cell suspensions were isolated by Ficoll-Hypaque gradient centrifugation. Cells (100 µL, at a final density of 2 × 10^5^ cells/well ) from Lewis rats were stimulated with 10 µg/mL IRBP in 96-well culture plate in triplicate in a humidified 5% CO_2_ atmosphere at 37°C for 48 h. MTT reagent (10 µL/well, at a final concentration of 5 g/L) was pulsed into each well 6 h before termination of culture. At the end of culture, the cells were centrifuged at 1000×g for 10 min and supernatants discarded. DMSO (150 µL) was added to each well and oscillated for 30 s to dissolve the formazan crystals. Optical density (OD) values were detected using a microplate reader (MK3, Lebo, Dutch) at a wavelength of 570 nm, and the results were presented as the average of triplicate absorbance.

### Semi-quantitative Reverse Transcription-polymerase Chain Reaction (RT-PCR)

Total RNA was extracted from the freshly isolated retinas of every group at the end of the experiment (day 14) using Trizol (Invitrogen, Carlsbad, USA) reagent according to the manufacturer’s protocol. The first strand of cDNA was synthesized using total RNA (1 µg/reaction), which was incubated at 70°C for 10 min and then chilled on ice immediately. Then 4 µL of 25 mmol/L MgCl_2_, 2 µL of reverse transcription buffer, 2 µL of 10 mmol/L dNTP mixture, 0.5 µL recombinant RNasin ribonuclease inhibitor, 15 U AMV reverse transcriptase, 0.5 µL oligo(dT) primer, and 8.4 µL nuclease-free water were added. The mixture was incubated at 42°C for 1 h, heated at 95°C for 5 min, and then incubated at 4°C for 5 min. cDNA (50–100 ng) was amplified using primers (Sango, Shanghai, China) specific for rat IL-17, IFN-γ, and β-actin. Primer sequences and optimal PCR annealing temperatures and cycle numbers are listed in Table 1. PCR was carried out for 35 cycles according to the following procedure: at 94°C for 40 s, annealing for 40 s, and at 72°C for 40 s. The PCR products were subjected to 1.5% agarose gel electrophoresis and semi-quantified by OD with an Image-Pro Plus software program (Ipwin 32, Medis Cybernetics, USA). RNA expression was quantified by comparison with the internal control β-actin.

**Table 1:** Primer sequences for rat genes and optimal PCR annealing temperatures and cycle number.

Gene	Annealing temperature, °C	Primers	Size, bp
IL-17	53.5	Forward: 5-CATGTGCCTGATGCTGTTGCT- 3	313 bp
		Reverse: 5- ATCTTCTCCACCCGGAAAGTG-3	
IFN-γ	61	Forward: 5- ATGGATGCTATGGAAGGAAAGA-3	369 bp
		Reverse: 5- GGCACACTCTCTACCCCAGAA-3	
β-actin	60	Forward: 5-GATATCGCTGCGCTCGTCGTC-3	350 bp
		Reverse: 5- GTCCCFFCCAGCCAGGTCCAG-3	

### Cytokine Assay

Serum was collected from every group at the end of the experiment. Cytokines IFN-gamma and IL-17 were measured using commercially available ELISA kit according to the manufacturer’s instructions. To assess the intraocular cytokine IL17, snap-frozen eyes were mechanically homogenized, resuspended in 1 ml PBS with proteinase inhibitors(Leupeptin(10 uM), Pepstatin A(1 uM) and phenylmethanesulfonyl fluoride(1 mM). sonicated and centrifuged at 12,000 rpm for 5 min. The supernatant was stored at –80°C until use.

### Flow Cytometric Analysis

On days 14 after immunization we investigated the frequency of IL17+T cells. Individual cell suspensions from the spleen and peripheral mononuclear blood cells (PBMC) were prepared. Aliquots of 1×10^6^ T cells were pre-cultured with Brefeldin A (BFA, 0.5 mg/ml; ENZO, USA), ionomycin (5 μg/ml; ENZO, USA), and phorbol-12-myristate-13-acetate (PMA, 50 ng/ml; ENZO, USA) for four hours before intracellular staining. After permeabilization, Th17 cells were stained with PE-labeled anti-mouse IL-17 antibodies (eBioscience, USA) and fluorescein isothiocyanate (FITC)-labeled anti-rat CD4 Abs (eBioscience, USA).

Data collection and analysis were performed with a flow cytometer (FACSCalibur; BD PharMingen) and appropriate software (CellQuest; BD PharMingen).

### Statistical Analysis

Experiments were performed at least two times. Disease severity for each animal was calculated as an average of both eyes. Data are presented as mean ± standard deviation (SD). EAU scores were compared by the Mann-Whitney U test. Continuous variables from the other experiments were analyzed with the unpaired Student’s t-test. P<0.05 was considered to be statistically significant. The mean values of treatment groups were compared with one-way ANOVA. When the ANOVA showed that there were significant differences (*P*<0.05) between the groups, a Dunnett’s test or Bonferroni’s test was used to identify the sources of these differences. This secondly obtained P value was considered statistically significant when *P*<0.05.

## Results

### EAU Model: Clinical Scoring

**Figure 1:**
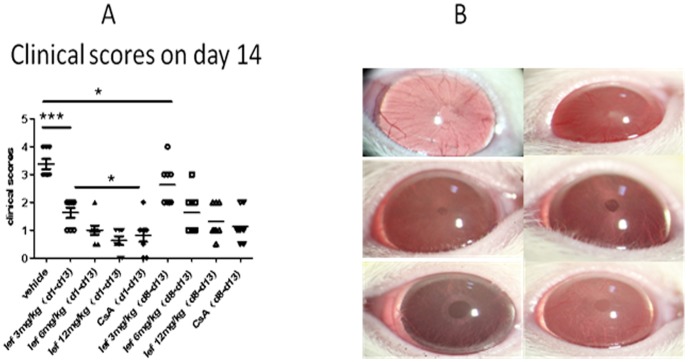
Clinical evaluation of EAU after immunization. (**A**) Rats were immunized with IRBP (d0) to induce uveitis and intragastric administration (d1–d13) with leflunomide (low dose, 3 mg/kg; medium dose, 6 mg/kg; high dose, 12 mg/kg) or CsA (10 mg/kg). At the time of oral administration, mean clinical scores were recorded (d13) and are represented as mean clinical scores ±SD. Data presented are the average of six to eight rats per group. Clinical scores were calculated by the nonparametric Mann-Whitney *U* test. Statistical significance compared with vehicle is indicated by asterisks (*P<0.05; **P<0.01; ***P<0.001). (**B**) The slit-lamp biomicroscopic of the eyes of different groups on day 14 after immunization. (a) Normal group rats eye showed normal iris vessels and red reflex. (b) Vehicle treated rats showed severe iris hyperemia and occlusion of pupil (c) leflunomide (low dose, 3 mg/kg). (d) leflunomide (medium dose, 6 mg/kg). (e) leflunomide (high dose, 12 mg/kg). (f) CsA (positive control, 10 mg/kg).

Daily oral administration of leflunomide prevented visible clinical signs of EAU development in comparison with vehicle treated controls. On day 14, vehicle group rat showed severe inflammation (mean clinical score, 3.4±0.5; n = 8.) There was a statistical difference in the mean clinical scores between the vehicle group and low dose group with leflunomide(mean clinical score, 1.6±0.5; n = 8, p<0.001) (Fig. 1A). All doses of leflunomide were effective at preventing clinically significant uveitis in a dose-dependent manner. The medium [6 mg/kg], and high [12 mg/kg] doses provided a greater protection than the low dose (Fig. 1A). More important, we observed dose-dependent effectiveness of leflunomide in animals that received gavage after disease onset (d8– d13; Fig. 1A). The leflunomide treatment led to a significant reduction of the clinical severity of EAU from day 14 after immunization compared to rats that received injections of physiological saline.

As shown in Figure 1B, most of the IRBP-treated rats shown severe illness developed including opaque anterior chamber obscured pupil(Fig. 1B.b). Peak clinical scores of 3 and 4 were observed on day 14. In group treated simultaneously with immunization, leflunomide orally strikingly reduced disease severity, and leflunomide treated rats showed only slight signs of anterior chamber inflammation especially the medium and high dose (Fig. 1B. d–e). In addition, the early clinical signs of EAU in the control group, including engorged blood vessels in the iris and abnormal pupil contraction, were primarily found from day 8, whereas in rats treated early with leflunomide, the onset of clinical EAU was delayed to day 11.

### EAU Model: Histopathology

**Figure 2:**
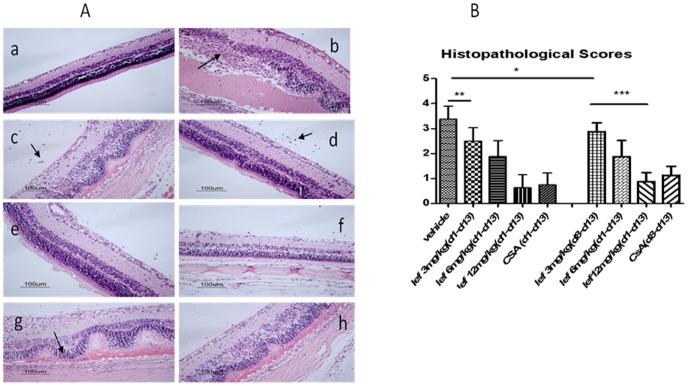
Histopathologic evaluation of EAU after immunization. (**A**) Representative histopathology of EAU in the different treatment groups. At the termination of the experiment, eyes were fixed and embedded in paraffin. Retinal tissue sections (×100) stained with hematoxylin and eosin. (a) the retina of a normal rat eye. (b) vehicle, physiological saline. (c) leflunomide (low dose, 3 mg/kg). (d) leflunomide (medium dose, 6 mg/kg). (e) leflunomide (high dose, 12 mg/kg). (f) CsA (positive control, 10 mg/kg). *Black arrows*: inflammatory cellular infiltrates surrounding retinal vessels and in the vitreous. (**B**) Effect of leflunomide on histopathology of rats with EAU. Histology scores in rats on day 14 after immunization of different groups showed significant histologic improvement in groups. Histology scores were calculated by the nonparametric Mann-Whitney *U* test. Values are expressed as mean ±SD. (*P<0.05; **P<0.01; ***P<0.001) (*n* = 8).

Masked evaluation of histopathology after experimental termination of in vivo experiments also revealed dose-dependent efficacy of leflunomide. Histopathology of eyes collected on day 14 revealed that vehicle-treated rats showed retinal disorganization; photoreceptor destruction and inflammatory cells in the vitreous, uvea, and retina (Fig. 2A.b). Leflunomide preventively treated rats had significantly milder pathologic severity of the retina compared to the untreated control rats (Fig. 2A. c–e). Extensive ocular inflammation and photoreceptor destruction was observed in eyes of the vehicle-treated rats (Fig. 2A.b), which had the highest combined anterior and posterior histopathologic scores. Retinas from vehicle-treated rats had marked inflammatory infiltration, focal destruction of the nuclear layers that were more prominent in the outer nuclear layer, and total loss of inner and outer photoreceptor segments. Compared with vehicle-treated controls, a significant reduction in the median EAU scores were observed in the eyes from the low, medium, and high leflunomide preventively treated rats (Fig. 2B). On day 14, when the disease was at its peak pathological score (approximately 3.4), leflunomide preventively treated rats given the low dose of leflunomide had a score of nearly 2.5, which was significantly lower (*P*<0.05) than control rats.

All animals in the low-dose leflunomide therapeutic treatment group exhibited uveitis, including retinal folds and small granuloma formation (Fig. 2A.g, black arrows). Retinal inflammatory cellular infiltration was obviously alleviated in medium-dose and high-dose leflunomide therapeutic treatment groups(Fig. 2A.h).

### Leflunomide Suppression of Lymphocyte Proliferation

**Figure 3:**
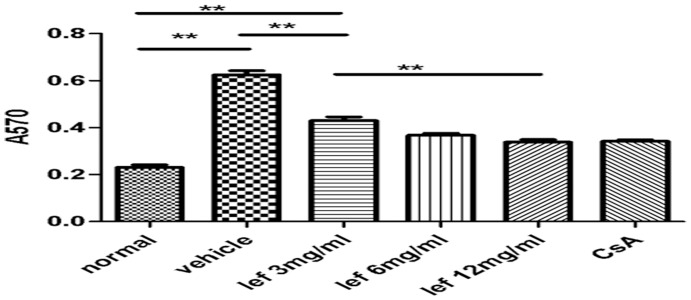
Proliferative response of splenocytes collected on day 14 following immunization and stimulated in vitro with IRBP-peptide (10 ug/ml). The proliferation of cultured cells was examined by MTT assay. Results are expressed as mean±SD (n = 8). *P<0.05, **P<0.01.

Along with suppression of intraocular inflammation, *in vitro* proliferation of lymphocytes determined from rats treated with leflunomide. Lymphocyte proliferation was significantly suppressed in cultures stimulated with IRBP (Fig. 3).

### Effects of Leflunomide on Chemokine mRNA Expression in the Retina

**Figure 4:**
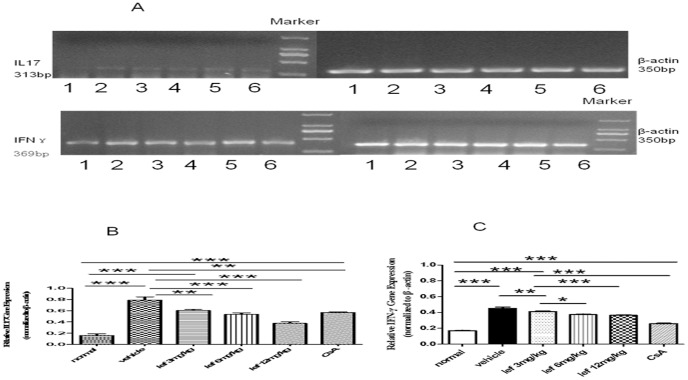
The effect of leflunomide on ocular IL17 mRNA and IFN-γmRNA expression in EAU rats. **** The mRNA levels were normalized to those of β-actin(Figure 4A). RT-PCR analysis reveals that the mRNA expression of IL17 and IFN-γwere significantly increased in IRBP-treated rats. In contrast, leflunomide treatment reduced the expression of IL17 and IFN-γin EAU rats(Figure 4B,4C). Data are expressed as the mean±SD (*n* = 8). *P<0.05; **P<0.01; ***P<0.001. 1, normal; 2, vehicle; 3, low-dose leflunomide (3 mg/kg); 4, medium-dose leflunomide (6 mg/kg); 5, high-dose leflunomide (12 mg/kg); 6, CsA (10 mg/kg).

As Fig. 4A shows, there was significant difference in the mRNA of IL17 and IFN-γ between the leflunomide-treated and the vehicle-treated eyes. The medium and high doses provided more statistically significant protection than the low dose. Thus, these data suggest that an orally administered leflunomide suppresses the mRNA expression of IL17 (Fig. 4B) and IFN-γ(Fig. 4C) in the eyes with ongoing ocular inflammation.

### Analysis Cytokines IFN-r and IL-17 Levels in EAU and Treated with Leflunomide

**Figure 5:**
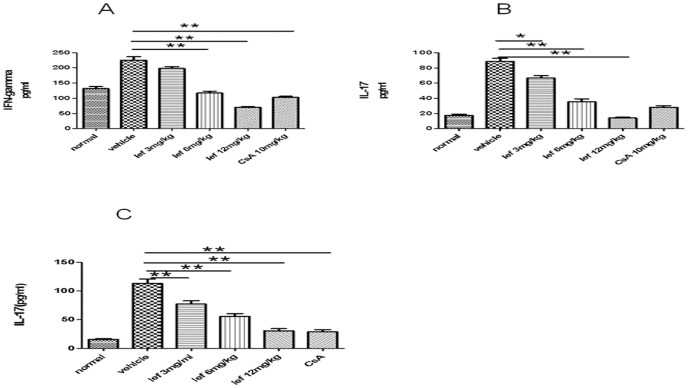
The expression levels of inflammatory cytokines IFN-gamma(Fig. 5A) and IL17(Fig. 5B) in the serum on day 14 were quantified by ELISA. leflunomide significantly decreased the serum production of IFN-gamma and IL17. Comparison between groups was made using one-way ANOVA; *P<0.05; **P<0.01; The level of IL17(Fig. 5C) in the eye on day 14 were quantified by ELISA. leflunomide significantly decreased ocular production of IL17. Comparison between groups was made using one-way ANOVA; *P<0.05; **P<0.01.

We examined whether oral administered of leflunomide suppressed the production of inflammatory cytokine, IL17 and IFN-γin the serum of immunized rats, IL17 in the eyes of immunised rats. As fig. 5 shows, on day 14, the production of IFN-γ (Fig. 5A) and IL17 (Fig. 5B) was markedly suppressed in the leflunomide-treated serum by ELISA (*P*<0.05). As Fig. 5C shows, on day 14, the production of IL17 was markedly suppressed in the leflunomide-treated eyes (*P*<0.05). These results suggest that leflunomide has the capacity to suppress inflammatory cytokine levels in serum and eyes with ongoing EAU.

### Detection of Th17 Cells in EAU and Effect of Leflunomide Blockade against Murine Th17 Cells

**Figure 6:**
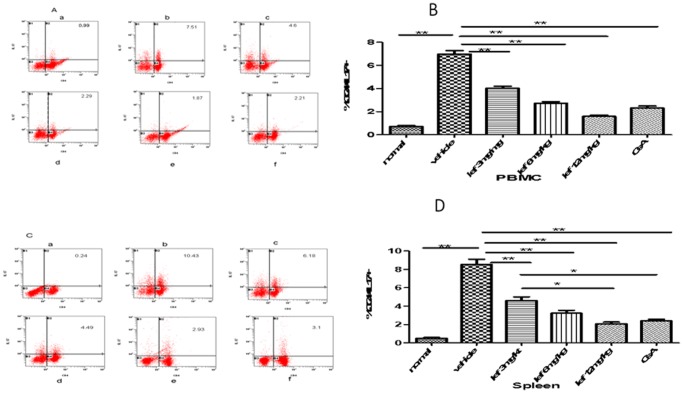
Detection of Th17 cells in preventive group. By flow cytometric analysis, fresh cells from PBMC (Fig. 6A–B), fresh cells from spleen(Fig. 6C–D). The numbers in the histograms indicate the percentages of cells that were double-positive for IL-17/CD4. Data are expressed as the mean±SD (*n* = 6). *P<0.05; **P<0.01; a, normal; b, vehicle; c, low-dose leflunomide (3 mg/kg); d, medium-dose leflunomide (6 mg/kg); e, high-dose leflunomide (12 mg/kg); f, CsA (10 mg/kg).

On day 14 after immunization, rats were sacrificed and spleen cells and PBMC were collected. As expected, the spleens and PBMC from EAU donors contained significant numbers of CD4+IL-17+Th17-type T cells. By contrast, fresh splenic and PBMC CD4+T cells from normal non-immunized rats had only a small population of IL-17+cells(Fig. 6A–6D). T cells from vehicle group produced large amounts of IL-17 in the presence of IRBP peptide in vitro(Fig. 6A, Fig. 6C). Rats showed a marked decreased in IL17+T cells in spleen and PBMC after treated with leflunomide. These results suggest that leflunomide may prevent the differentiation of Th17 cells in EAU.

## Discussion

This study clearly demonstrates that oral administration of leflunomide was capable of suppressing the ongoing development of autoimmune uveoretinitis in the eye. CsA and FK-506 (tacrolimus) have been previously determined to be effective in preventing the development of uveitis in the EAU rodent model [27], [28]. Leflunomide is a novel immunosuppressive agent that also acts through calcineurin inhibition [29].

In the present study, we observed that rats treated with the leflunomide were capable of suppressing the ongoing process of autoimmune uveoretinitis in the eye. In addition, we show that oral leflunomide was capable of suppressing the ongoing process of ocular inflammation in the rat model of EAU. Leflunomide was effective in preventing the development of severe, clinical EAU with doses as low as 3 mg/kg when administered daily starting from the day of immunization with IRBP. Furthermore, the retinal detachment, photoreceptor layer damage, edema, and severe infiltration of leukocytes in the posterior chamber and other pathologic markers of EAU were markedly suppressed in the leflunomide-treated rats. The amelioration of intraocular inflammation, supported by histopathologic evidence of this effect, suggests that leflunomide may be a useful suppression for ocular inflammatory conditions.

However, the mechanism to suppress ocular inflammation and the effect for systemic immune response remain unknown. With the recognition of Th17 immunoregulation [30], [31], the understanding of involvement of Th17 in ocular diseases like uveitis [32], [33] is increasing. Beside conventional immune cells, the contribution of resident tissue cells to the modulation of IL17 must not be overlooked. In this study, to elucidate the anti-inflammatory mechanism of the oral leflunomide, we measured the concentrations of inflammatory cytokines such as IFN-γand IL17. As shown, IL17 production was inhibited in the leflunomide preventive group eyes on day 14 after immunisation. Furthermore, both the level of IFN-γand IL17 were decreased in the leflunomide preventive groups serum on day 14 after immunisation. Taken together, our results indicate that oral leflunomide has the capacity to suppress effector inflammatory cells via inhibition of the inflammatory cytokine expression in the ocular tissue and serum.

CsA was used as the positive control because of its proven efficacy in preventing EAU formation and treating established EAU in the rodent model [34], [35], [36]. CsA was able to inhibit clinical symptom of uveitis and to decrease clinical signs of uveitis. Based on the clinical and histopathologic findings in this study, the therapeutic efficacy of leflunomide appears equal to cyclosporine.

The *in vitro* effects of leflunomide on lymphocytes suggest that its efficacy may be mediated through the inhibition of T-cell effector responses. Lymphocytes harvested from animals treated with leflunomide demonstrated significantly decreased antigen proliferation compared with untreated control animals. This finding was observed with the intragastric administration of 3 mg/kg/d dosing and also with higher doses. Leflunomide also demonstrated a dose-dependent ability to inhibit the proliferation of stimulated lymphocytes.

Th1 and Th17 cells are involved in autoimmune disorders. Pro-inflammatory Th1 and Th17 immune responses have been previously shown to be salient features of uveitis and scleritis [37], [38]. Th17 cells constitute a third subset of effector helper T cells. The effector functions of Th17 cells are distinct from those of Th1 and Th2 cells [7], [39], [40]. Th17 cells and IL-17 play a critical role in the pathogenic mechanisms of intraocular inflammation in an animal model of human uveitis [7], [41], [42]. In humans, the number of Th17 lymphocytes increases during acute uveitis or scleritis and decreases after treatment [32], [43], [44], [45], [46]. Indeed, EAU in rats is suppressed by anti–IL17 antibody [8]. In the present study, we showed that fresh intraocular T cells from immunized EAU donors had a large population of IL-17+cells, suggesting that CD4+IL-17+Th17-type T cells may be associated with the pathogenic mechanisms of intraocular inflammation. Importantly, spleen and PBMC CD4+EAU T cells produced less IL-17 if the rats were treated with leflunomide. Thus, leflunomide provides protection from inflammation by Th17-type helper T cells in EAU. This provides evidence that IL-17 plays a crucial role in EAU and that leflunomide reduces EAU severity by reducing Th17 responses. Our results indicate that oral leflunomide suppressed IL17 gene expression and IFN-γgene expression in the eyes of EAU rats. The decreased production of both Th1 and Th17 lymphocyte subsets, as evidenced by the decreased production of both IFN-gamma and IL17, can be explained by the decreased severity of EAU in rats pre-treated by leflunomide. The ability of leflunomide to downregulate pro-inflammatory Th1 and Th17 effector responses, combined with its efficacy in the prevention EAU. Leflunomide, an isoxazole derivative that is structurally unrelated to other immunomodulatory agents, interferes in multiple immunological pathways. Further investigations are needed to clarify the mechanism of leflunomide effect on immune cells. The dosage of leflunomide in rats used in the experiment was generally well-tolerated [14], [19], [47]. All experimental rats in leflunomide-treated group survived the experiment. We observed leflunomide side effects mostly in the high dose group such as weight loss and hairs sparseness.

In summary, we have demonstrated that oral leflunomide diminishes ocular inflammation by inhibiting pathogenic T-cell expansion and cytokine production in EAU. The protective effect of leflunomide on the development of EAU suggests that leflunomide might be an attractive potential therapeutic approach for the treatment of autoimmune disease.
